# ctGAN: combined transformation of gene expression and survival data with generative adversarial network

**DOI:** 10.1093/bib/bbae325

**Published:** 2024-07-09

**Authors:** Jaeyoon Kim, Junhee Seok

**Affiliations:** School of Electrical and Computer Engineering, Korea University, 145 Anam-ro, Seongbuk-gu, Seoul, 02841, Korea; School of Electrical and Computer Engineering, Korea University, 145 Anam-ro, Seongbuk-gu, Seoul, 02841, Korea

**Keywords:** style-transfer deep generative model, generative adversarial network, gene expression, survival analysis

## Abstract

Recent studies have extensively used deep learning algorithms to analyze gene expression to predict disease diagnosis, treatment effectiveness, and survival outcomes. Survival analysis studies on diseases with high mortality rates, such as cancer, are indispensable. However, deep learning models are plagued by overfitting owing to the limited sample size relative to the large number of genes. Consequently, the latest style-transfer deep generative models have been implemented to generate gene expression data. However, these models are limited in their applicability for clinical purposes because they generate only transcriptomic data. Therefore, this study proposes ctGAN, which enables the combined transformation of gene expression and survival data using a generative adversarial network (GAN). ctGAN improves survival analysis by augmenting data through style transformations between breast cancer and 11 other cancer types. We evaluated the concordance index (C-index) enhancements compared with previous models to demonstrate its superiority. Performance improvements were observed in nine of the 11 cancer types. Moreover, ctGAN outperformed previous models in seven out of the 11 cancer types, with colon adenocarcinoma (COAD) exhibiting the most significant improvement (median C-index increase of ~15.70%). Furthermore, integrating the generated COAD enhanced the log-rank *p*-value (0.041) compared with using only the real COAD (*p*-value = 0.797). Based on the data distribution, we demonstrated that the model generated highly plausible data. In clustering evaluation, ctGAN exhibited the highest performance in most cases (89.62%). These findings suggest that ctGAN can be meaningfully utilized to predict disease progression and select personalized treatments in the medical field.

## Introduction

With increasing interest in artificial intelligence (AI), research using machine learning and deep learning models has garnered significant attention across various domains [1–4]. In biomedicine, these models have been extensively investigated for applications in medical imaging [5–7], genome sequencing [8–12], and the understanding of protein structure and properties [13–16]. Several studies have used electronic health records for clinical and research purposes, extracting keywords, and diagnosing diseases using natural language processing [17–19].

Recently, deep learning algorithms have been extensively used to investigate gene expression for disease diagnosis, treatment, and survival analysis. Numerous studies have aimed to identify genes with the greatest relevance to specific diseases and use them to predict patient prognosis [20–23]. Additionally, deep learning algorithms can predict patient prognosis based on diverse treatment methods and anticipate disease progression, including metastasis [24, 25]. Survival analysis research on diseases with high fatality rates, such as cancer, is being actively conducted [26, 27].

Gene expression has been actively studied for several decades, resulting in the accumulation of large-scale datasets such as The Cancer Genome Atlas (TCGA) and Gene Expression Omnibus [28–30]. However, the size of these datasets is insufficient for training deep learning models. Advanced deep learning studies typically use tens of thousands of samples; for instance, MNIST comprises 60 000 images and CIFAR-10 includes 50 000 images for training. By contrast, in the TCGA dataset, the maximum number of samples for a specific cancer type is ~1000. Although substantial data are crucial, the collection of an unlimited number of samples from individuals is not feasible and incurs high costs for measuring the expression of a single gene. These challenges hinder the implementation of deep learning models. Owing to the numerous genes and relatively small sample size, overfitting problems frequently arise during training [31]. Nevertheless, gene expression analyses can provide new insights.

To address this problem, recent studies have focused on transforming gene expression data across species, for instance, by leveraging genomic data recorded from human and mouse models to develop statistical methods such as EBT and mEBT [32, 33]. These models aim to identify genes with high response concordance to overcome the challenges posed by discrepancies between animal and human responses. In addition, several studies have developed deep generative models for generating plausible gene expression data using style transformation. Representative examples of deep generative models include variational autoencoders (VAE) [34, 35] and generative adversarial networks (GANs) [36, 37]. Various models have been developed for specific applications. For instance, Lotfollahi et al. [38] presented trVAE, and Russkikh et al. [39] proposed stVAE, both of which implement style-transfer methodologies in conditional VAE (CVAE) to generate gene expression. trVAE overcomes the limitations of CVAE using the maximum mean discrepancy in the decoder layer. It demonstrates high robustness and accuracy in predicting the conditions and classes for images and single-cell RNA-seq data. stVAE utilizes CVAE, Y-autoencoders, and adversarial feature decomposition for RNA-seq data harmonization using technical factors or biological details as style components to facilitate style transfer. It exhibits high performance in terms of style and semantic prediction accuracy.

Style transformation techniques not only enable the prediction of experimental outcomes, which, in turn, reduces the time, cost, and risk associated with data acquisition, but also enhance model accuracy when sufficient data is available. However, both models exclusively concentrate on generating transcriptomic data, resulting in reduced clinical utility. To be applicable to clinical purposes, the generation should be extended to include clinical outcomes. Therefore, we developed a style-transfer method capable of generating both gene expression and clinical data to enhance survival analysis. Instead of using conditional generation, we leveraged a cyclic generation model for data augmentation through style transformation between breast cancer and 11 other cancer types. Training a cyclic-generation model is more challenging than training a conditional model. However, we addressed this issue by modifying the internal structure of the generator and discriminator to better fit our data and by employing a training technique that enhanced training stability.

We showed that the accuracy of survival analysis was not merely owing to an increase in the quantity of data. Using visualization tools, we demonstrated the distribution of the data, ensuring that the generated and reconstructed data closely approximated the real data. Additionally, in the clustering evaluation, ctGAN demonstrated the highest performance in most cases. Our approach enables the reflection of varying survival times for different cancer types, thereby enhancing the effectiveness of the survival analysis. Furthermore, ctGAN outperforms existing methods.

## Material and methods

### Model architecture

A deep generative style-transfer architecture called ctGAN is proposed to enhance survival analysis through gene expression and survival data augmentation. Gene expression and survival data were obtained from TCGA, which encompasses 33 different cancer types. In this study, we focused on 12 specific types of cancer, including breast invasive carcinoma (BRCA). Among these 12 cancer types, BRCA had the highest sample count, with 1051 samples, and the second-highest sample count was 530 samples; however, this was still insufficient for conducting survival analysis.

This discrepancy between the high number of genes and the limited sample size presents a significant challenge when applying deep learning methodologies. A deep learning model exists that automatically selects genes during training using various regularization techniques [40]. However, this approach does not resolve the fundamental issue of an insufficient sample size. Therefore, we utilized a style-transfer methodology to augment the sample sizes by transforming gene expression and survival data between different cancer types. Our experimental results demonstrate that this approach is beneficial for cancer survival analysis.

**Figure 1:**
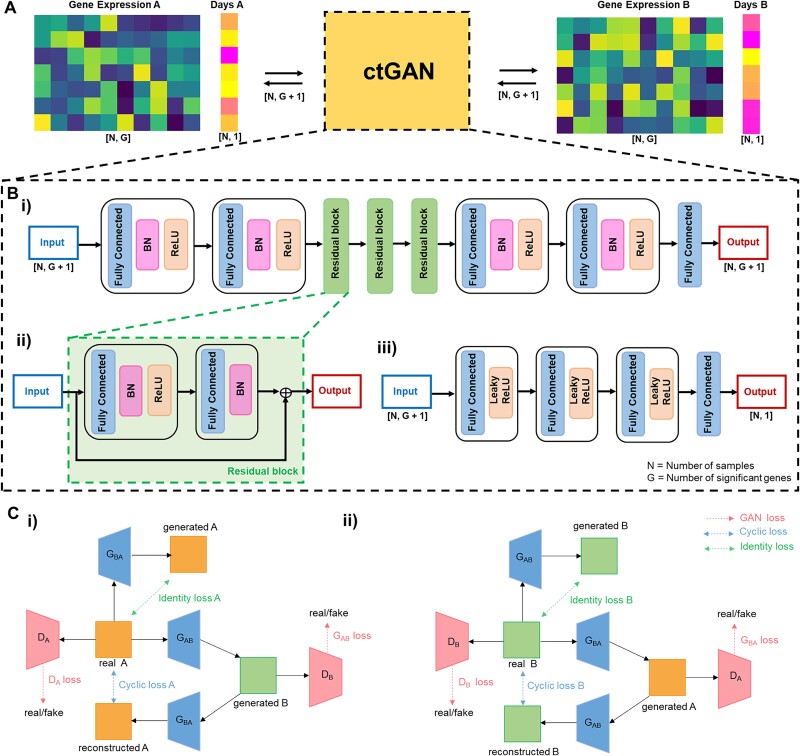
ctGAN architecture and underlying mechanisms. (A) ctGAN transforms gene expression and survival time data across different types of cancer. (B) i) generator structure of ctGAN. ii) structure of the residual block within the generator of ctGAN. iii) discriminator structure of ctGAN. (C) Cyclic-consistent adversarial network scheme. i) Transformation from cancer type A to cancer type B. ii) transformation from cancer type B to cancer type A.

Because BRCA has the largest dataset among the 12 cancer types, we conducted transformations between BRCA and the other 11 cancer types. Consequently, as shown in Fig. 1A, new gene expression and survival time data were generated for all 11 cancer types, matching the sample count of BRCA. Moreover, BRCA itself received newly transformed gene expression and survival time data from the 11 other cancer types.

As shown in Fig. 1C, cyclic-consistent GAN [41–44] involves the training of two generators, ${G}_{AB}:A\to B$ and ${G}_{BA}:B\to A$, along with two discriminators, ${D}_A$ and ${D}_B$. Fig. 1C (i) illustrates the transformation of data from domain A to domain B, whereas Fig. 1C (ii) shows the transformation from domain B to domain A. A fundamental concept in cyclic domain translation is cycle consistency: for each sample $a\in A$ that undergoes a transformation from domain A to domain B and then returns to domain A, it should remain identical to the original sample, i.e. $a\to{G}_{AB}(a)\to{G}_{BA}\left({G}_{AB}(a)\right)\approx a.$ Similarly, for each sample $b\in B$, $b\to{G}_{BA}(b)\to{G}_{AB}\left({G}_{BA}(b)\right)\approx b$. Additionally, the concept of identity loss indicates that for each sample $b\in B$ that is fed into ${G}_{AB}$ as input, the output should be identical to the original sample, i.e. $b\to{G}_{AB}(b)\approx b$. Therefore, cycle-consistent adversarial networks have three types of losses: GAN, cyclic, and identity losses.

For the GAN loss, we applied a least squares loss instead of a negative log-likelihood objective to stabilize the model training procedure as follows:


(1) \begin{align*} {L}_{GAN}\left({G}_{AB},{D}_B,A,B\right)=&\ {\mathbb{E}}_{b\sim{p}_{data}(b)}\left[{\left({D}_B(b)-1\right)}^2\right]\nonumber\\&+{\mathbb{E}}_{a\sim{p}_{data}(a)}\left[{\left({D}_B\left({G}_{AB}\Big(a\right)\right)}^2\right] \end{align*}

In the case of cyclic and identity losses, we applied the L1 norm as follows:


(2) \begin{align*} {L}_{Cycle}\left({G}_{AB},{G}_{BA}\ \right)=&\ {\mathbb{E}}_{a\sim{p}_{data}(a)}\left[{\left\Vert{G}_{BA}\left({G}_{AB}(a)\right)-a\right\Vert}_1\right]\nonumber\\ &+{\mathbb{E}}_{b\sim{p}_{data}(b)}\left[{\left\Vert{G}_{AB}\left({G}_{BA}(b)\right)-b\right\Vert}_1\right] \end{align*}

(3) \begin{align*} {L}_{Identity}\left({G}_{AB},{G}_{BA}\ \right)=&\ {\mathbb{E}}_{b\sim{p}_{data}(b)}\left[{\left\Vert{G}_{AB}(b)-b\right\Vert}_1\right]\nonumber\\ &+{\mathbb{E}}_{a\sim{p}_{data}(a)}\left[{\left\Vert{G}_{BA}(a)-a\right\Vert}_1\right]\qquad\qquad \end{align*}

Therefore, our full objective is calculated as follows:


(4) \begin{align*} L\left({G}_{AB},{G}_{BA},{D}_A,{D}_B\right)=&\ {L}_{GAN}\left({G}_{AB},{D}_B,A,B\right)+{L}_{GAN}\left({G}_{BA},{D}_A,B,A\right)\nonumber\\ & + {L}_{Cycle}\left({G}_{AB},{G}_{BA}\ \right)+{L}_{Identity}\left({G}_{AB},{G}_{BA}\ \right) \end{align*}

Because the original domain-transfer GAN was initially designed for style transfer in image data, we modified the internal architecture of the generator and discriminator to make it applicable to gene expression. Skip connections and fully connected layers were incorporated into the generator of ctGAN to achieve an effect similar to that of the ResNet architecture [45]. As shown in Fig. 1B (ii), the residual block comprised two hidden layers. The first hidden layer included one fully connected layer [46], batch normalization [47], and ReLU activation [48], whereas the second hidden layer comprised one fully connected layer and batch normalization. As shown in Fig. 1B (i), the generator was composed of two hidden layers, each comprising a single fully connected layer, batch normalization, and ReLU activation, followed by three residual blocks, two additional hidden layers, and a fully connected layer.

As shown in Fig. 1B (iii), the discriminator comprised three hidden layers, each containing one fully connected layer and leaky ReLU activation [49], culminating in the final fully connected layer.

### Model training and parameter setting

Training and fine-tuning cyclic-consistent GAN models using conventional cross-validation methods may be challenging due to the simultaneous training of the generator and discriminator. Because adjusting one model can affect the other, accomplishing an adequate balance is critical [50, 51]. A discriminator loss converging to near zero suggests that the discriminator learns faster than the generator, hindering proper generator training. Additionally, the generator depends heavily on cyclic and identity losses during training, resulting in insufficient learning of the GAN loss. To address this issue, we adjusted the training method, enabling the generator to undergo three times more epochs than the discriminator, and repeated the training three times on the same dataset [52]. Regarding the loss functions, we used the mean square error (MSE) loss for the GAN loss and L1 loss for the cyclic and identity losses.

Regarding parameter settings, we assessed the model’s performance in terms of concordance index (C-index) [53] (see Supplementary Methods) enhancement and analyzed data distribution using t-SNE and clustering evaluation metrics. The detailed hyper-parameter results depicted in Fig. S1 elucidate the effects of varying the number of hidden nodes of the Generator and Discriminator.

### Survival analysis method

To evaluate survival analysis, we used supervised principal component analysis (SuperPC), which is a widely used method for survival analysis based on gene expression. SuperPC was introduced by Bair et al. [54] and is particularly beneficial when dealing with high-dimensional data, wherein the number of features significantly outweighs the number of samples. This method closely resembles traditional principal component analysis except that it utilizes a subset of predictors chosen based on their correlation with the outcome. This method is applicable to both regression and generalized regression problems, including survival analysis. Using SuperPC, we assessed the improvement in the C-index using ctGAN. SuperPC was implemented in Python.

### Dataset

The multi-omics data utilized in this research involved RNA-sequencing data representing mRNA expression. Gene expression and survival data were collected from TCGA, which included 33 different cancer types. Among these, only samples labeled as tumors were extracted and matched with survival data using sample IDs. After excluding cases with a survival time of 0 days, we selected only those cancers with 300 or more samples and at least 50 instances wherein the event occurred. Within the context of survival analysis, an event corresponds to either an occurrence of the outcome of interest or a censoring point indicating that the outcome had not occurred by the end of the study. Twelve cancers were included in this study (Table 1).

**Table 1:** Cancer abbreviations, full names, total sample counts, and event occurrence counts for the twelve types of cancer considered in the study

Cancer abbreviation	Cancer name	#Sample	#Event observed
BLCA	Bladder urothelial carcinoma	395	110
BRCA	Breast invasive carcinoma	1051	106
COAD	Colon adenocarcinoma	366	51
HNSC	Head and neck squamous cell carcinoma	498	167
KIRC	Kidney renal clear cell carcinoma	530	158
LGG	Brain lower grade glioma	523	100
LIHC	Liver hepatocellular carcinoma	340	89
LUAD	Lung adenocarcinoma	500	123
LUSC	Lung squamous cell carcinoma	474	153
OV	Ovarian serous cystadenocarcinoma	379	207
SKCM	Skin cutaneous melanoma	439	154
STAD	Stomach adenocarcinoma	333	71

### Gene selection

The raw gene expression data comprises 60 660 ensemble IDs. However, not all genes are significant for survival analysis. Therefore, if the goal of data augmentation is to improve survival analysis, genes relevant to survival analysis should be chosen. For instance, when augmenting colon adenocarcinoma (COAD) data using ctGAN, it is essential to select genes related to COAD survival analysis. Therefore, we calculated the C-index for each gene using the Cox proportional-hazards (CoxPH) model. During this process, we obtained 60 660 C-index values, with higher values indicating genes that were more closely related to COAD survival. Consequently, we selected the top 300 genes and used the same set of genes as in the BRCA data as well. The selected gene sets for data augmentation through style transfer varied depending on the targeted cancer type. The experimental results, including those obtained when varying the number of genes used or randomly removing genes, are depicted in Figs S2 and S3. Additionally, Fig. S4 shows the outcomes of the experiment integrating significant genes from both the targeted and source cancer (BRCA), which reveal that employing only significant genes from the targeted cancer yields better performance.

### Overview of style transfer workflow

**Figure 2:**
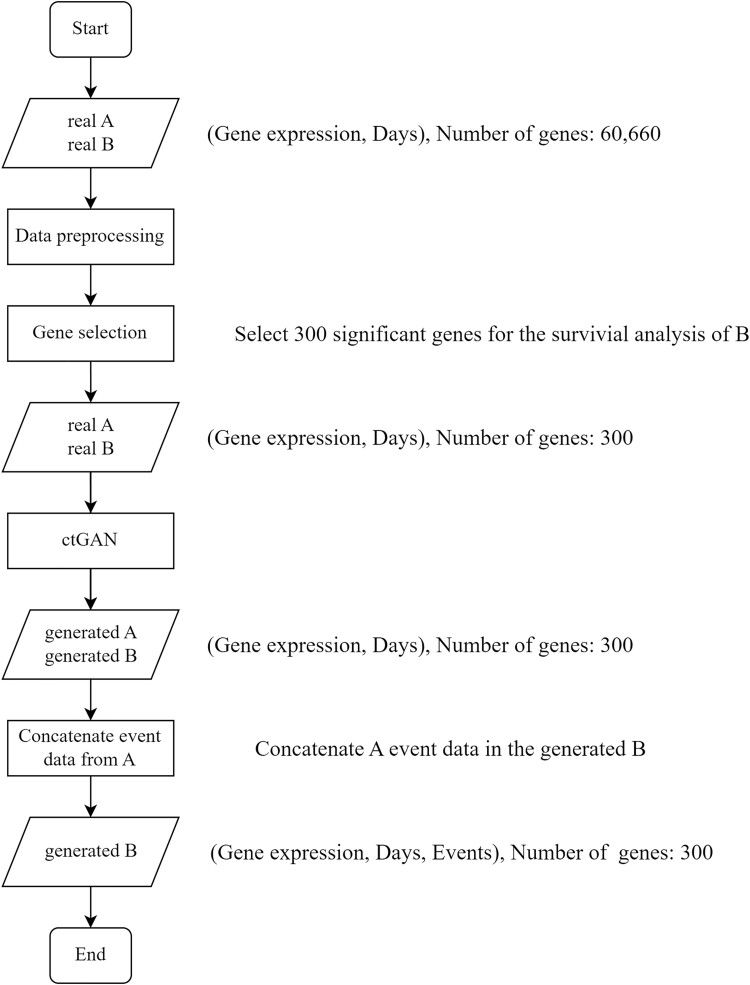
Flowchart of the style transfer process, which comprises data preprocessing, gene selection, model training, and data generation.

Figure 2 presents an overview of the style transfer workflow from cancer types A to B. Data preprocessing was performed on gene expression and survival data from real datasets A and B, which exclusively contained tumor samples. The preprocessing involved the removal of samples with 0 survival days and log-normalization with ${X}_{new}:={\log}_2\left(X+1\right)$, where $X$ is the expression value of the normalized fragments per kilobase transcript per million mapped reads, and survival time. Next, the top 300 significant genes relevant to the survival analysis of B were selected based on the gene expression of both A and B.

Consequently, 300-dimensional gene expression and survival time data for both A and B were fed as input into ctGAN, which, in turn, generated new gene expression and survival time data. Subsequently, by concatenating the real A event data with the generated B data, complete survival data were created. The generated B data were then used in the survival analysis alongside the real B data. Here, if we presume A as BRCA and B as COAD, ctGAN will generate gene expression and survival time data for 366 BRCA and 1051 COAD samples, because the original sample size of BRCA was 1051 and COAD was 366.

Further details regarding the architecture and training of trVAE and stVAE, along with a comparison to ctGAN, can be found in the Supplementary Methods section and Figs S5 and S6.

## Results

### Evaluation of survival analysis enhancement

ctGAN has been suggested to enhance survival analysis by transforming gene expression and survival data. The performance of the ctGAN was evaluated and compared to that of existing methods: trVAE and stVAE. The C-index and log-rank *p*-value were used as evaluation metrics for survival analysis. The C-index is among the most frequently used metrics for survival analysis. It does not evaluate the exact survival time of a subject but rather compares the survival times of multiple subjects [53]. The log-rank *p*-value indicates the significance of the two groups when plotted on the Kaplan–Meier (KM) curve as high- and low-risk groups [55]. A *p*-value less than 0.05 is considered statistically significant.

**Figure 3:**
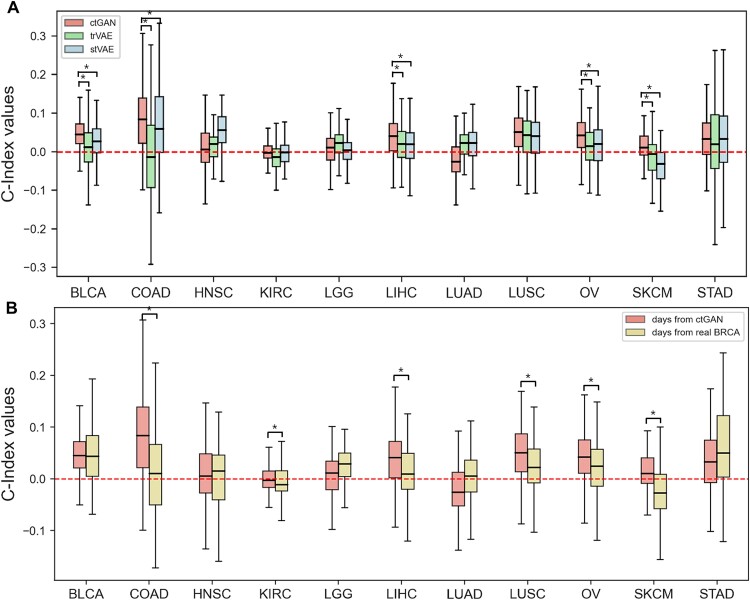
C-index improvement across 11 types of cancer. (A) Comparison of C-index enhancements among ctGAN, trVAE, and stVAE. (B) Comparison of enhancement in C-index between generating gene expression and survival time using ctGAN versus generating only gene expression while retaining the original survival time from BRCA. The performance estimation in both (A) and (B) were measured with 100 cross-validation iterations. The dash line in both (A) and (B) represents the scenario where only real data were used (^*^p-value <0.05 when the ctGAN achieves the best performance).

To illustrate the enhanced accuracy of survival analysis achieved with the generated data, we initially divided the real dataset into training and test sets. Subsequently, the data generated by ctGAN was integrated into the training set. We then trained the SuperPC model and evaluated its performance using the C-index on the test set data. Given the variability of the C-index depending on the dataset, we conducted 100 rounds of cross-validation by partitioning the real data into different random state numbers. As shown in Fig. 3A, based on the median value from these 100 rounds of cross-validation, ctGAN exhibited improved C-index values in nine out of 11 cancer types, excluding KIRC and LUAD. For those nine types of cancer, except for HNSC and LGG, the analysis including generated samples exhibits statistically significant differences (*p*-value <0.05) when compared to using only real data. Notably, the model exhibited the most significant performance enhancement for COAD (median C-index value increased from 0.656 for only real samples to 0.759 when including generated samples), representing a performance improvement of ~15.70%. In addition, ctGAN outperformed the other methods in seven cancer types, excluding HNSC, KIRC, LGG, and LUAD. Among these, the analysis of five cancer types (BLCA, COAD, LIHC, OV, SKCM) using ctGAN displayed statistically significant differences (*p*-value <0.05) compared to both trVAE and stVAE. This result confirms the robustness of ctGAN, indicating that the model performed well across various cancer datasets.

Conversely, compared to other models, trVAE yielded the highest performance improvement for only two cancers (LGG, LUAD), with only LUAD demonstrating statistical significance (*p*-value <0.05) compared to both ctGAN and stVAE. Similarly, stVAE exhibited the highest performance improvement for only two cancers (HNSC, KIRC), but none of these two cases resulted in statistical significance (*p*-value <0.05) when compared to both ctGAN and trVAE. These results confirm that ctGAN achieved greater performance enhancement and statistical significance than trVAE and stVAE. Fig. S7 depicts an additional experiment exploring the C-index improvement when only generated data are used.

ctGAN generated gene expression and survival time data through style transfer between BRCA and 11 other types of cancer. Alternatively, gene expression was generated using ctGAN, while survival time data were taken directly from BRCA. As shown in Fig. 3B, the performance was notably higher in seven cancers (excluding HNSC, LGG, LUAD, and STAD) when the survival time data were also generated through ctGAN. Among these, the analysis of six cancer types (COAD, KIRC, LIHC, LUSC, OV, SKCM) revealed statistically significant differences (*p*-value <0.05). The proposed method considered diverse survival times across different types of cancer, thereby improving the efficiency of survival analysis.

**Figure 4:**
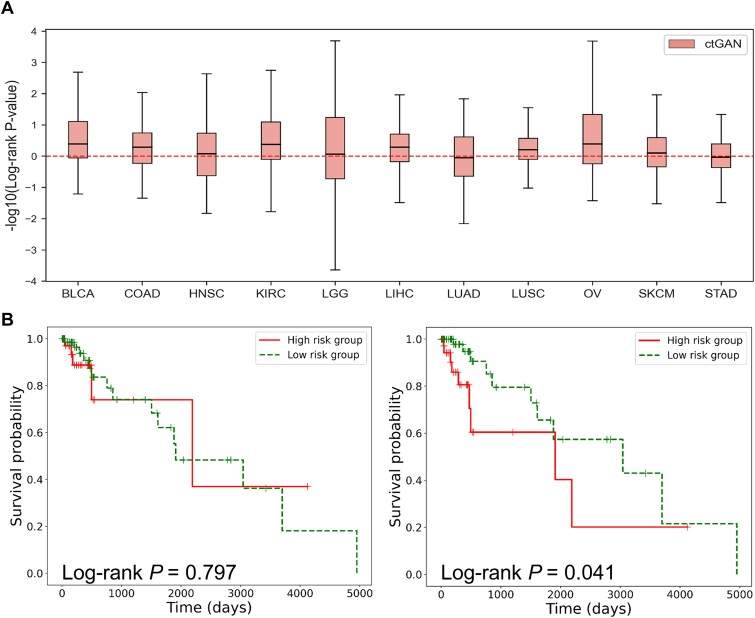
KM plot and log-rank test *p*-value. (A) Augmenting real samples with those generated by ctGAN shows enhanced log-rank test *p*-values across 11 cancer types, as indicated by -log10(log-rank *p*-value). Estimation performances were measured with 100 iterations of cross-validations. The dash line represents the scenario where only real data were used. (B) KM plot using only real COAD data. (C) KM plot using both real COAD and generated COAD data by ctGAN.

As shown in Fig. 4A, considering the median value from 100 rounds of cross-validation, ctGAN demonstrated enhanced log-rank *p*-values in nine of the 11 cancer types, excluding LUAD and STAD.

In Fig. 4B and C, the KM plots are presented for COAD (the cancer type for which the model showed the most substantial performance improvement, Fig. 3A). Owing to the impracticality of presenting KM plots for all 100 cross-validation results, a split of ~7:3 was conducted in chronological order to create the training and testing sets. The SuperPC model was trained on the training set. If the partial hazard in the test set exceeded the median partial hazard in the training set, the group was classified as high-risk; otherwise, it was classified as low-risk.

Fig. 4B shows the KM plot when using only real COAD data, whereas Fig. 4C shows the KM plot when real COAD data were combined with the COAD generated by ctGAN. The generated COAD data were merged into the training set for SuperPC model training, and a KM plot was generated using the test set. The results revealed a considerably more significant log-rank *p*-value of 0.041 when integrating the generated COAD data compared to a *p*-value of 0.797 when using only the real COAD data. This outcome underscores the effectiveness of style transformation in gene expression and survival data using ctGAN when other clinical conditions remain unchanged.

We conducted additional experiments for COAD. Employing Random Survival Forests instead of SuperPC led to a statistically significantly (*p*-value <0.002) better C-index than using only real data. Additionally, training ctGAN without identity loss improved performance compared to using only real data. However, the median C-index exhibited a statistically significant (*p*-value <0.001) decreased of 8.43% (from 0.759 with identity loss to 0.695 without identity loss).

### Style transfer validation

The style transfer performance of ctGAN was evaluated using visualization tools to examine data distributions. Our emphasis was on COAD, for which the model exhibited the highest performance improvement among the 11 cancer types (Fig. 3A).

Using ctGAN, COAD was transformed into BRCA, and vice versa. A successful style transfer between the two domains implied that the generated data closely resembled the real data from the same domain. In other words, the generated COAD data should be close to the real COAD data, and the generated BRCA data should be adjacent to the real BRCA data. Additionally, the distribution of the reconstructed data must be inspected. Reconstructed data refers to instances wherein the data were transformed into another domain and then returned to their original domain. For instance, reconstructed COAD signifies data that were initially real COAD, transformed into BRCA, and then returned to COAD. Similarly, the reconstructed BRCA represents the data that were initially real BRCA, transformed into COAD, and then reverted to its original BRCA state. The closer the reconstructed data matched the real data, the more effective the style transfer by ctGAN.

Using ctGAN, we can predict the gene expression and survival time of patients with breast cancer that has transformed into colon cancer. Naturally, if a patient’s data are transformed back into breast cancer data, retaining the original data is expected to yield accurate results.

**Figure 5:**
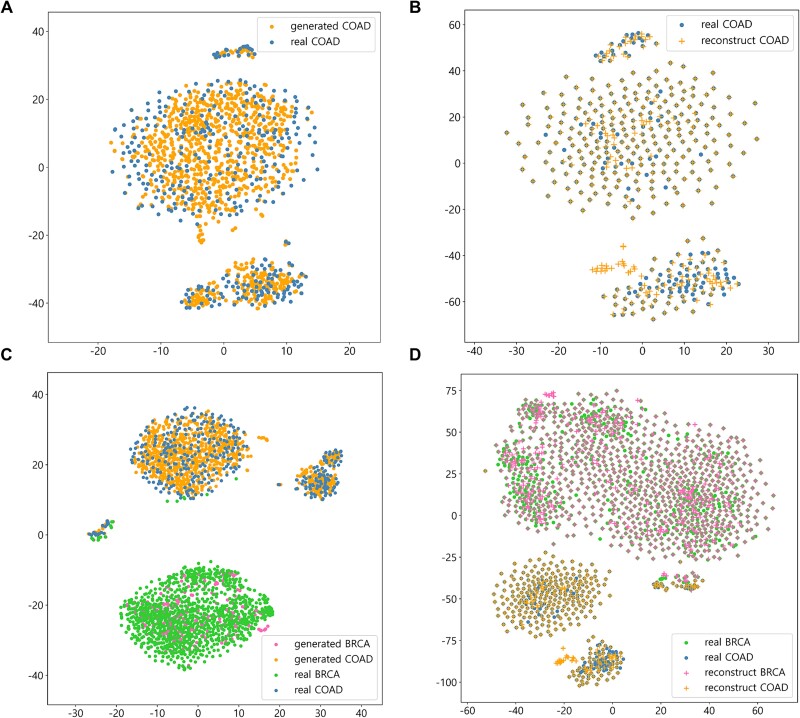
Visualization of real, generated, and reconstructed samples using t-SNE for COAD and BRCA gene expression. (A) Real and ctGAN-generated gene expressions for COAD visualized with t-SNE perplexity set to 50. (B) Real and ctGAN-reconstructed gene expressions for COAD visualized with a t-SNE perplexity set to 50. (C) Real and ctGAN-generated gene expressions for BRCA and COAD visualized with a t-SNE perplexity set to 50. (D) Real and ctGAN-reconstructed gene expressions for BRCA and COAD visualized with a t-SNE perplexity set to 50.

As shown in Fig. 5A and C, the generated data closely approximated the real data, confirming effective clustering between BRCA and COAD. Additionally, Fig. 5B and D illustrate that the reconstructed data closely resembled the real data, indicating successful clustering between BRCA and COAD.

**Figure 6:**
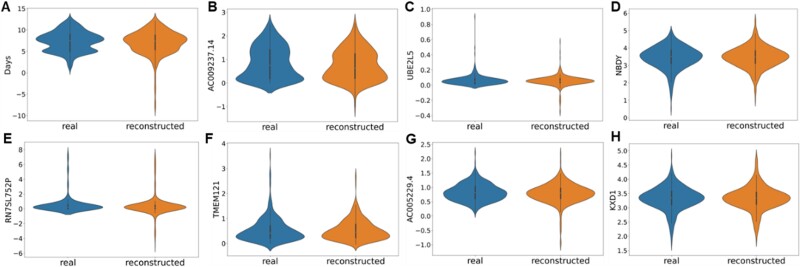
Violin plot representing the distribution of real data and reconstructed data by ctGAN for the top seven significant genes in COAD survival analysis, including survival times.

As shown in Fig. 6, the data distribution of the real and reconstructed COAD for survival time and the top seven significant genes were remarkably similar. Figures 5 and 6 indicate that the improvement in survival analysis performance by ctGAN was not a result of random data augmentation. Instead, the model generated highly plausible data, which exerted a positive influence on survival analysis.

Table 2 presents a comparative analysis of the clustering evaluation metrics, MSE and R-squared (R2) values, for the proposed model and other style transfer-based frameworks across all 11 types of cancers.

**Table 2:** Comparison of performance metrics between various style transfer-based frameworks and the proposed model. The highest performance values are in bold, and second highest performance values are in italics

Cancer Model	BLCA	COAD	HNSC	KIRC	LGG	LIHC	LUAD	LUSC	OV	SKCM	STAD
**None**											
KNN purity	0.947	0.979	*0.972*	0.992	**1**	*0.996*	0.989	0.973	0.997	0.969	**1**
NMI	0.691	0.864	0.726	0.952	**1**	*0.983*	0.925	0.535	0.984	0.494	**1**
ARI	0.809	0.929	0.795	0.977	**1**	*0.994*	0.961	0.566	0.994	0.482	**1**
Silhouette	0.532	0.628	0.428	**0.681**	**0.689**	*0.734*	0.607	0.585	0.73	0.444	*0.711*
Dunn index	0.773	*0.952*	*0.558*	0.966	*1.028*	*1.168*	0.871	0.855	1.211	**0.636**	*1.13*
MSE	NA	NA	NA	NA	NA	NA	NA	NA	NA	NA	NA
R2	NA	NA	NA	NA	NA	NA	NA	NA	NA	NA	NA
**Ours**											
KNN purity	**0.987**	*0.988*	**0.983**	**0.999**	**1**	**0.999**	**0.996**	*0.988*	**1**	*0.985*	*0.999*
NMI	*0.803*	**0.93**	**0.874**	**0.992**	**1**	**0.992**	**0.971**	**0.934**	**0.996**	**0.919**	*0.992*
ARI	*0.864*	**0.961**	**0.918**	**0.997**	**1**	**0.997**	**0.988**	**0.967**	**0.999**	**0.955**	*0.997*
Silhouette	*0.613*	*0.654*	0.432	0.663	*0.687*	0.694	**0.69**	**0.656**	*0.735*	*0.487*	**0.722**
Dunn index	*0.782*	0.713	0.553	*0.972*	**1.036**	0.558	**1.19**	**1.027**	*1.286*	0.6	**1.235**
MSE	**0.079**	*0.204*	*0.55*	**0.068**	**0.075**	**0.072**	**0.141**	**0.059**	**0.067**	**0.09**	0.295
R2	**0.828**	**0.678**	*0.56*	**0.868**	**0.841**	**0.791**	**0.775**	**0.841**	**0.801**	**0.884**	**0.589**
**stVAE**											
KNN purity	0.973	0.986	0.94	0.995	*0.999*	**0.999**	0.992	0.958	*0.999*	0.97	0.99
NMI	**0.863**	0.888	0.753	0.972	*0.996*	**0.992**	*0.955*	0.719	*0.989*	*0.877*	0.929
ARI	**0.923**	0.936	0.806	0.987	*0.999*	**0.997**	*0.978*	0.793	*0.996*	*0.93*	0.964
Silhouette	**0.636**	**0.693**	*0.459*	*0.677*	0.676	**0.743**	0.637	*0.631*	**0.739**	0.445	0.697
Dunn index	**0.924**	**1.02**	0.553	**1.055**	0.89	**1.418**	*0.972*	*0.868*	**1.374**	0.574	1.084
MSE	*0.183*	**0.165**	**0.219**	*0.226*	*0.242*	*0.231*	*0.267*	*0.185*	*0.189*	*0.201*	**0.163**
R2	*0.616*	*0.61*	**0.626**	*0.627*	*0.567*	*0.439*	*0.593*	*0.536*	*0.455*	*0.738*	*0.446*
**trVAE**											
KNN purity	0.964	0.986	0.967	0.991	0.995	0.991	0.988	0.973	0.983	**0.986**	0.99
NMI	0.755	*0.912*	*0.798*	0.94	0.951	0.939	0.914	*0.87*	0.858	0.868	0.908
ARI	0.814	*0.953*	*0.877*	0.972	0.975	0.971	0.957	*0.929*	0.904	0.92	0.947
Silhouette	0.518	0.512	0.417	0.539	0.572	0.623	0.583	0.577	0.562	0.436	0.544
Dunn index	0.284	0.333	0.27	0.303	0.337	0.341	0.324	0.321	0.294	0.287	0.281
MSE	0.387	0.314	0.579	0.43	0.356	0.33	0.552	0.382	0.329	0.63	*0.251*
R2	0.254	0.297	0.254	0.369	0.432	0.214	0.223	0.163	0.084	0.466	0.176

The k-nearest neighbor (KNN) purity [56], normalized mutual information (NMI) [57], and adjusted random index (ARI) [58] are clustering evaluation metrics applicable when the class labels are known. If the cancer type is the same, it should have the same class label. These metrics assess the alignment of the clustering results with the actual labels. The values range from 0 to 1, with a value closer to 1 indicating better clustering performance.

Conversely, the Silhouette and Dunn indices are clustering evaluation metrics suitable when class labels are unknown [59, 60]. The Silhouette index, ranging from −1 to 1, increases with higher intra-cluster similarity and lower inter-cluster similarity. A value closer to 1 indicates a higher clustering performance. However, the Dunn index increases with a smaller maximum intra-cluster distance and a larger minimum inter-cluster distance. The Silhouette index only considers the similarity with neighboring clusters, whereas the Dunn index considers the distances between all clusters. When computing the NMI, ARI, Silhouette index, and Dunn index, a Gaussian mixture was used as the clustering model [61].

Successful style transfer models can enhance clustering performance compared to using only real data, with minimal errors between the real and reconstructed data. In Table 2, ‘None’ indicates the use of only real data, resulting in NA values for both MSE and R2 owing to the absence of reconstructed data.

As shown in Table 2, ctGAN exhibited the highest performance, followed by stVAE. In particular, ctGAN consistently achieved the highest or second-best results across various metrics. Of the 77 evaluation items, the highest result accounted for 64.94% with 50 instances, and the second highest result comprised 19 instances (24.68%). Together, they constituted 89.62% of the entire set.

For trVAE, KNN purity, NMI, and ARI values increased for certain cancers. However, in most cases, the Silhouette and Dunn indices exhibited a decline in performance. Notably, trVAE exhibited larger MSE and small R2 values compared with those of ctGAN and stVAE. Moreover, as shown in Fig. 3A, although trVAE improved the C-index through data augmentation, it could not be considered that the data were well generated.

### ctGAN with SCAN-B dataset

To validate the effectiveness of ctGAN with datasets other than TCGA, we conducted experiments on the SCAN-B dataset [62], which comprises RNA-seq data from breast cancer patients. Here, the aim is to enhance survival analysis accuracy for TCGA-BRCA through data augmentation. Therefore, we generated TCGA-BRCA data from two cancers with the largest samples sizes among 11 other TCGA cancers: KIRA and the second largest, LGG. We trained two distinct models, one using the real TCGA-BRCA dataset and another combining TCGA-BRCA with augmented data. Subsequently, we utilized SCAN-B data as the test set to evaluate the C-index. The results indicate that using only the real TCGA-BRCA dataset yielded a C-index of 0.643, whereas combining data from KIRC increased the C-index to 0.66, and further enhancement was observed by incorporating data from LGG, with a C-index of 0.668.

**Figure 7:**
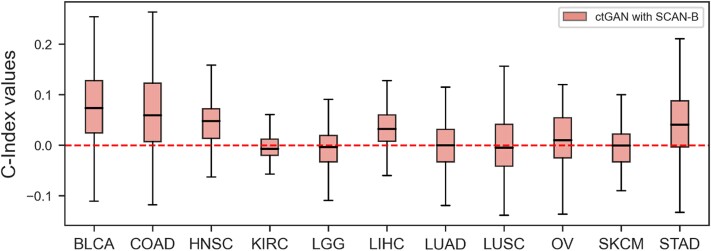
Improvement in C-index across 11 types of cancer when using ctGAN on the SCAN-B dataset.

Additionally, we used SCAN-B data as the source domain instead of TCGA-BRCA data, thereby generating 11 TCGA cancer gene expression datasets using the SCAN-B dataset. Fig. 7 shows that the C-index values improved for seven out of the 11 cancer types, excluding KIRC, LGG, LUSC, SKCM.

## Conclusion

This study proposed ctGAN, a deep generative model using the TCGA dataset, to combine transformations of gene expression and survival data with GAN. Challenges in implementing deep learning models, including overfitting, arise because of the limited sample size relative to the large number of genes. Although previous methods, trVAE and stVAE, attempted to address this issue, their applicability for clinical purposes is limited, as they generate only gene expression. By contrast, ctGAN enhanced cancer survival analysis by augmenting data via a style transformation between BRCA and 11 other cancers. We assessed the improvement in the C-index by comparing it with that of previous models, demonstrating the superior performance of ctGAN. ctGAN enhanced the C-index values in nine of the 11 cancer types, and outperformed previous models in seven of the 11 cancer types. Notably, the model exhibited a substantial improvement in performance for COAD, with the median C-index value increasing by ~15.70%. Furthermore, the integration of the generated COAD data resulted in a significantly lower log-rank *p*-value (0.041) compared with using only the real COAD data (*p*-value = 0.797). Using visualization tools, we observed that the generated and reconstructed data distributions closely approximated the real data. Additionally, in the comparative analysis of the clustering evaluation metrics, MSE and R2 values, ctGAN consistently achieved either the highest or second-best results across various metrics (89.62%).

Our method aims to address the challenge of insufficient biomedical research data by providing significant assistance and innovation. Subsequent predictive models are likely to demonstrate increased reliability, accuracy, and robustness by generating gene expression and survival data for effective deep learning model training. Furthermore, we anticipate that ctGAN can significantly contribute to the medical field by assisting in the prediction of disease progression and the selection of personalized treatments, thereby generating substantial clinical impact. For instance, by capturing the differences between various cancers, it could help predict the likelihood of a breast cancer patient developing other types of cancer and their expected responses to different treatments.

**Key Points**

- This study proposes a style-transfer deep generative model, ctGAN, to address existing challenges in the implementation of deep learning models for analyzing gene expression.
- Previous models have limited applicability for clinical purposes, but ctGAN enables the combined transformation of gene expression and survival data and improves survival analysis by augmenting data through style transformations.
- ctGAN demonstrates high plausibility in data generation based on distribution and clustering evaluations. The proposed method may enable predictions regarding the likelihood of a patient with breast cancer developing other types of cancer and responding differently to various treatment methods.

## Supplementary Material

20240620_BIB_SupplementaryFile_bbae325

## Data Availability

Codes have been deposited in GitHub https://github.com/jyoonkim/ctgan

## Author Biographies

**Jaeyoon Kim** is a Ph.D. candidate at Korea University. Her research interests include deep learning and machine learning, and their applications to bioinformatics, materials design, and cyber security.

**Junhee Seok** is a professor at Korea University. His research interests include deep learning, machine learning, statistics, reinforcement learning, and their applications to bioinformatics, materials design, natural language processing, and cyber security.
