# Supplementary material for: ctGAN: combined transformation of gene expression and survival data with generative adversarial network
Source: Brief Bioinform. 2024 Jul 9;25(4):bbae325. doi: 10.1093/bib/bbae325 (PMC11232285; doi:10.1093/bib/bbae325)
Supplement: 20240620_BIB_SupplementaryFile_bbae325 [file 20240620_bib_supplementaryfile_bbae325.docx]

**Supplementary Methods**

**C-index**

The C-index is the most common and accurate index used in survival analysis. Rather than assessing the exact survival time of an individual, it compares the survival times among multiple subjects. In other words, it gauges the ability to predict the death sequence.

The concordance probability between two subjects is defined by the following equation:

|  | $c=\Pr\left( \hat{y_{1}}> \hat{y_{2}} \vert y_{1}\geq y_{2} \right)$ | (1) |
| --- | --- | --- |

Based on the above definition, the C-index can be computed as follows:

|  | $\hat{c}=\frac{1}{N_{p}}\sum_{m:C_{m}=1} \sum_{j:y_{m}<y_{j}} I\left[ S\left( \hat{y_{m}} \right\vert X_{m})<S\left( \hat{y_{j}} \right\vert X_{j}) \right]$ | (2) |
| --- | --- | --- |

where $S$ represents the survival function, I is an indicator function that counts cases when a given condition is met, $X_{m}$ denotes the features of instance $m$, and $y_{m}$ represents the time point at which an event occurs for subject $m$. This computation determines the proportion of all subject pairs evaluated wherein the survival function of subject $j$, who survived longer than subject $m$, is predicted to be greater, yielding a value between 0 and 1. $C_{m}$ = 1 indicates an event occurrence for subject $m$. Conversely, if subject $m$ was censored before the event occurred, it is excluded from the comparison because the longer survival of subject $j$ cannot be confidently determined. $N_{p}$ denotes the number of comparable pairs where an event occurred for subject $m$.

**Parameter setting**

Setting the number of hidden nodes of the generator and discriminator to 512 and 64, respectively, ensures that the generator undergoes more extensive training. The Adam optimizer was applied with a learning rate of 0.0002 for the generator and 0.0001 for the discriminator. For the generator loss, equal weights of 1 were assigned to the GAN, cyclic, and identity losses. The minibatch size was set to 16, and the number of training epochs was set to 500. The training-to-test set ratio was 9:1.

**Architecture and training of other frameworks**

We demonstrated the superiority of ctGAN by comparing its experimental results with those obtained from trVAE and stVAE. trVAE was implemented by adapting the code available at https://github.com/theislab/trvaep to our dataset, defining the input dimension as 301 and the number of classes as 2. Default settings were used for all other hyperparameters. stVAE was implemented using the code available at https://github.com/NRshka/stvae-source, setting the input dimension and the number of genes to 301, and the number of classes and labels to 2. All other hyperparameters maintained their default settings.

**Hyper-parameter tuning**


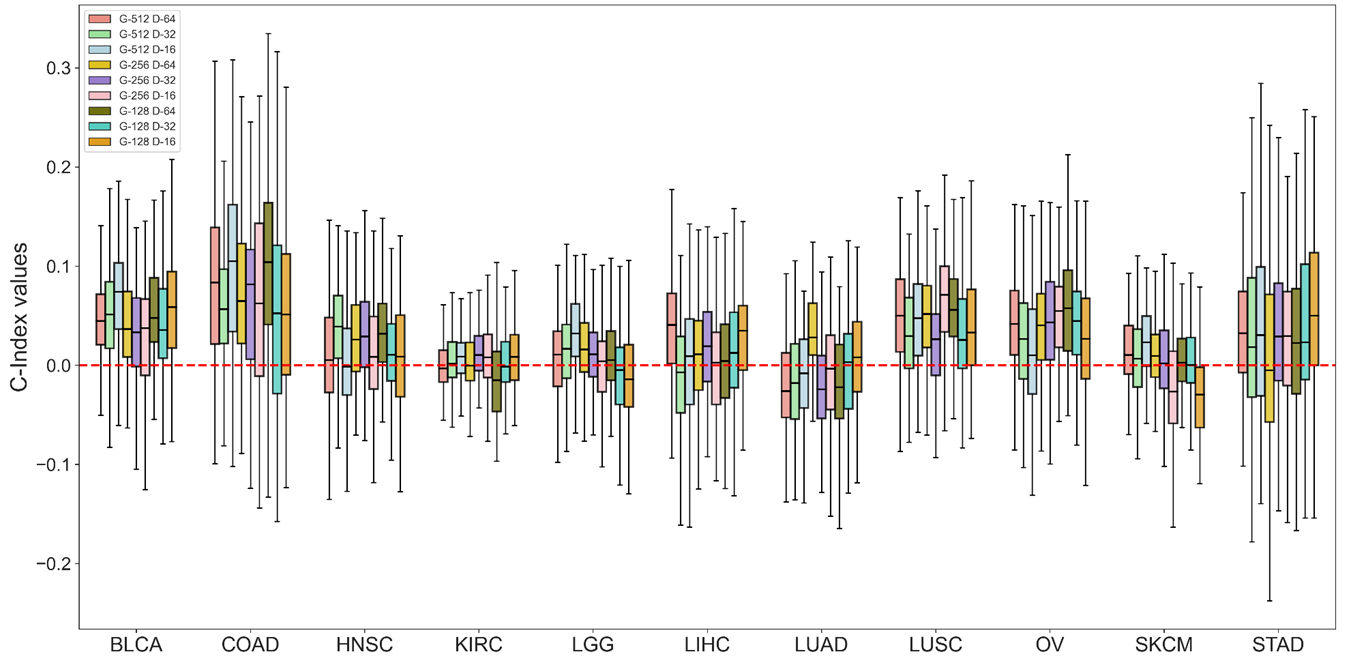


**Figure S1.** Improvement in C-index for different number of hidden nodes in the generator and discriminator across 11 cancer types.

To assess the improvement in the C-index after data augmentation with respect to using solely real data, we conducted experiments using various configurations of hidden nodes for the generator (G) and discriminator (D) during training. The number of genes was fixed at 300 for all experiments. When G-512 D-64, the C-index improved for 9 cancer types (excluding KIRC and LUAD), achieving its highest performance for LIHC. When G-512 D-32, the C-index improved for 9 cancer types (excluding LIHC and LUAD), accomplishing its highest performance for HNSC. When G-512 D-16, the C-index improved for 9 cancer types (excluding HNSC and LUAD), with the highest performance corresponding to BLCA, COAD, LGG, and SKCM). Similarly, when G-256 D-64, the C-index improved for all 9 cancer types (except for KIRC and STAD), with the highest performance corresponding to LUAD. For G-256 D-32, the C-index improved for 10 cancer types (excluding LUAD), with the highest performance yielded by KIRC. When G-256 D-16, the C-index improved for 9 cancer types (excluding LUAD and SKCM), with LUSC exhibiting the highest performance. When G-128 D-64, the C-index improved for 9 cancer types (excluding KIRC and LUAD), with the highest performance achieved for OV. When G-128 D-32, the C-index improved for 9 cancer types (excluding KIRC and LGG). When G-128 D-16, the C-index improved for 9 cancer types (excluding LGG and SKCM), with the highest performance exhibited by STAD.

Overall, the optimal number of hidden nodes varies depending on the cancer type. However, data augmentation through ctGAN generally contributed to improved survival analysis performance. This confirms the model’s robustness and its ability to be adjusted to optimal parameters based on the specific cancer data.


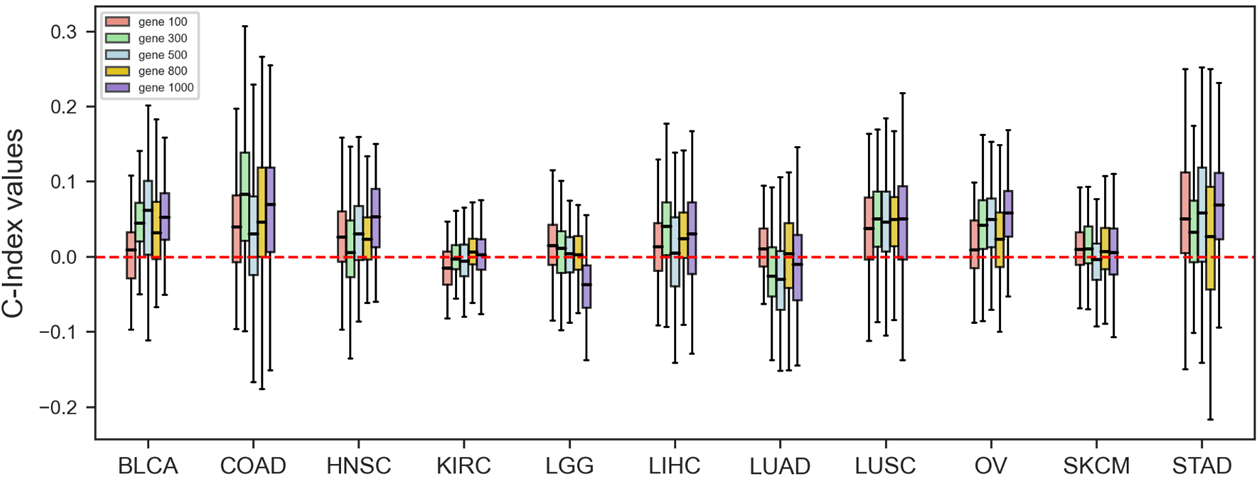


**Figure S2.** Improvement in C-index with variations in the number of genes across 11 cancer types.

We conducted experiments by training models with varying numbers of genes: 100, 300, 500, 800, and 1000. The number of hidden nodes was fixed at 512 in the generator and 64 in the discriminator. We assessed the improvement in C-index values after data augmentation compared to using only real data. With 100 genes, the C-index improved for 10 cancer types (excluding KIRC), showing its best performance for LGG and LUAD. With 300 genes, the C-index improved for 9 cancer types (excluding KIRC and LUAD) and performed the best for COAD, LIHC, and SKCM. With 500 genes, the C-index improved for 8 cancers (excluding KIRC, LUAD, and SKCM), with the highest performance accomplished with BLCA. With 800 genes, the C-index improved for all 11 cancer types, with the highest performance observed for KIRC. With 1000 genes, the C-index improved for 9 cancer types (excluding LGG and LUAD), with the highest performance observed for HNSC, LUSC, OV, and STAD.

Overall, the optimal number of genes varied depending on the cancer type. However, data augmentation through ctGAN generally enhanced survival analysis performance, indicating the model’s robustness.

**Random gene removal**


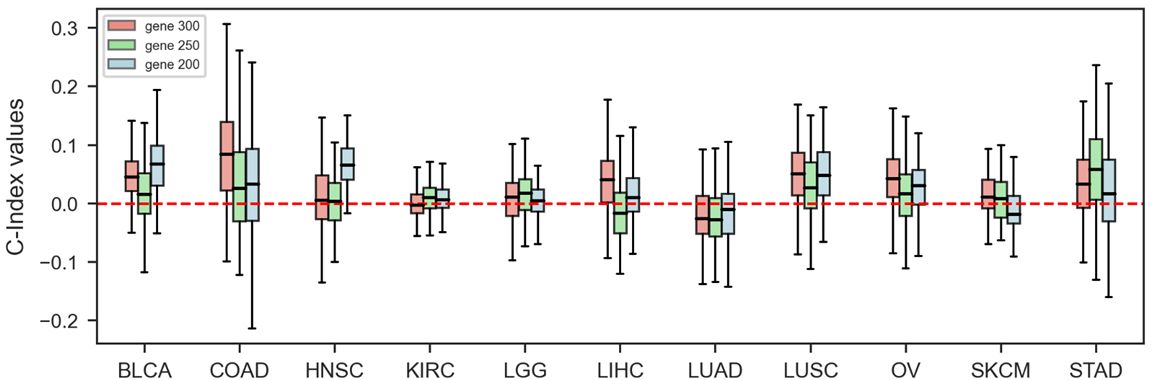


**Figure S3.** Improvement in C-index due to random gene removal across 11 cancer types.

We conducted an experiment that involved randomly removing 50 and 100 genes from a set of 300 significant genes for survival analysis. The results showed that survival analysis improved for 9 out of 11 cancer types in all cases (300, 250, and 200 genes). Specifically, when using the original 300 genes selected, the best performance was obtained for COAD, LIHC, LUSC, OV, and SKCM. When removing 50 genes (250 genes total), the best performance was observed for KIRC, LGG, and STAD. Finally, when removing 100 genes (200 genes total), the best performance corresponded to BLCA, HNSC, and LUAD.

Although the optimal number of genes varied depending on the cancer type and no single condition was optimal in all cases, the performance of ctGAN remained stable, confirming the model’s robustness.

**Gene selection domain**


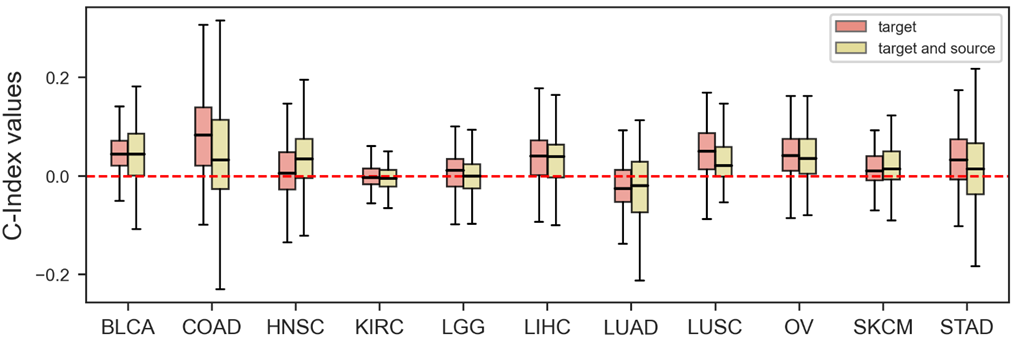


**Figure S4.** Improvement in C-index for different gene domain selections across 11 cancer types.

When using only significant genes from the target cancer, ctGAN exhibited improved C-index values for 9 out of 11 cancer types (excluding KIRC and LUAD). When integrating significant genes from both the target and source (BRCA) cancers, C-index values improved for 8 out of 11 cancers (excluding KIRC, LGG, and LUAD). Moreover, using only significant genes from the target cancer generally led to better performance than using significant genes from both the target and source cancers. Specifically, except for HNSC, LUAD, and SKCM, the remaining 8 out of the 11 cancer types demonstrated superior performance.

**trVAE and stVAE**

**
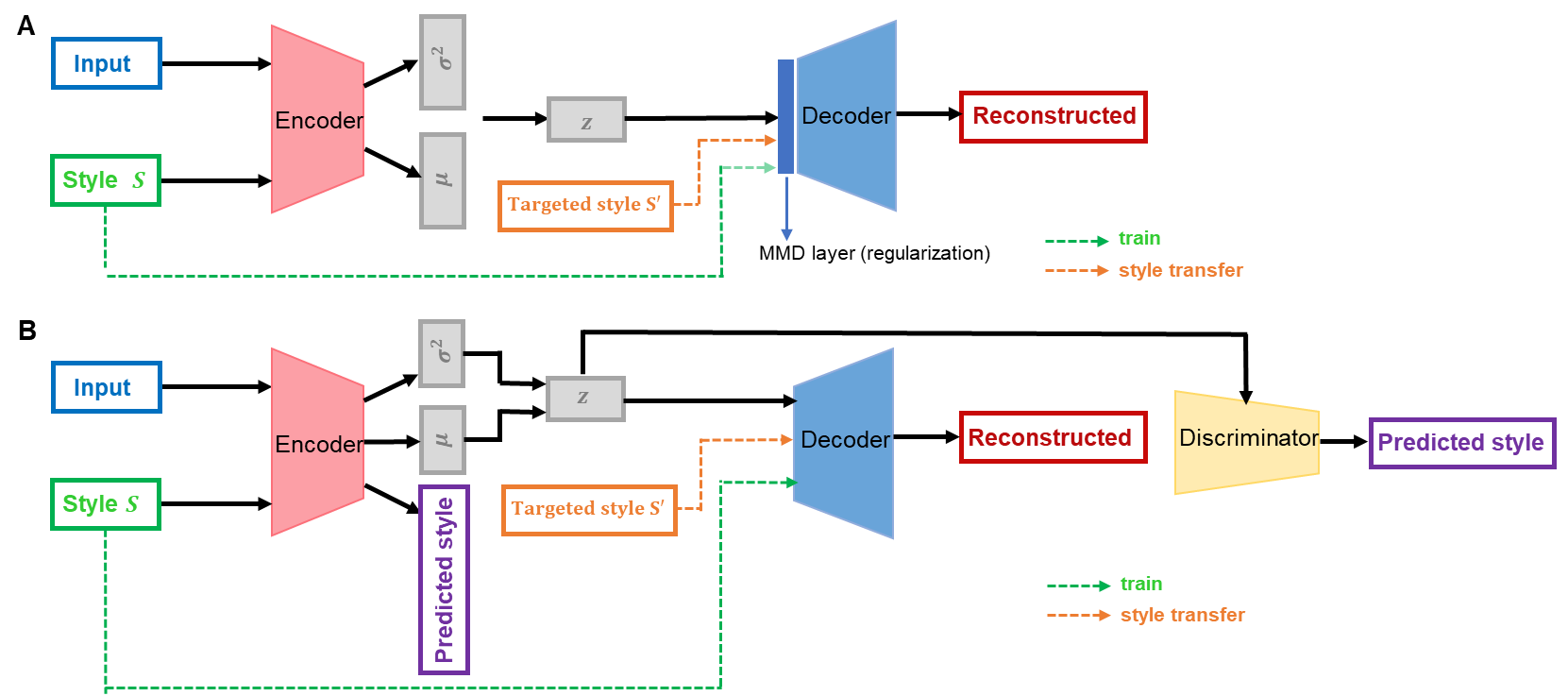
**

**Figure S5.** Architectures of (A) trVAE and (B) stVAE

As shown in Figure S5, both trVAE and stVAE incorporate a conditional variational autoencoder (CVAE). To overcome the limitation of CVAE, the first layer of decoder in trVAE is a maximum-mean discrepancy (MMD) layer that matches distributions across style conditions through regularization. Conversely, stVAE uses a Y-autoencoder and a discriminator to eliminate the information of style $S$ from the latent vector. The discriminator and encoder are trained under an adversarial learning scheme, the former to predict the style condition and the latter to effectively separate style information, hindering the discriminator’s style prediction. In both trVAE and stVAE, the decoder is trained to reconstruct the input data with the original style $S$. During style transformation, the decoder generates data that matches the targeted style $S'$.

**Comparison of ctGAN, trVAE, and stVAE**

**
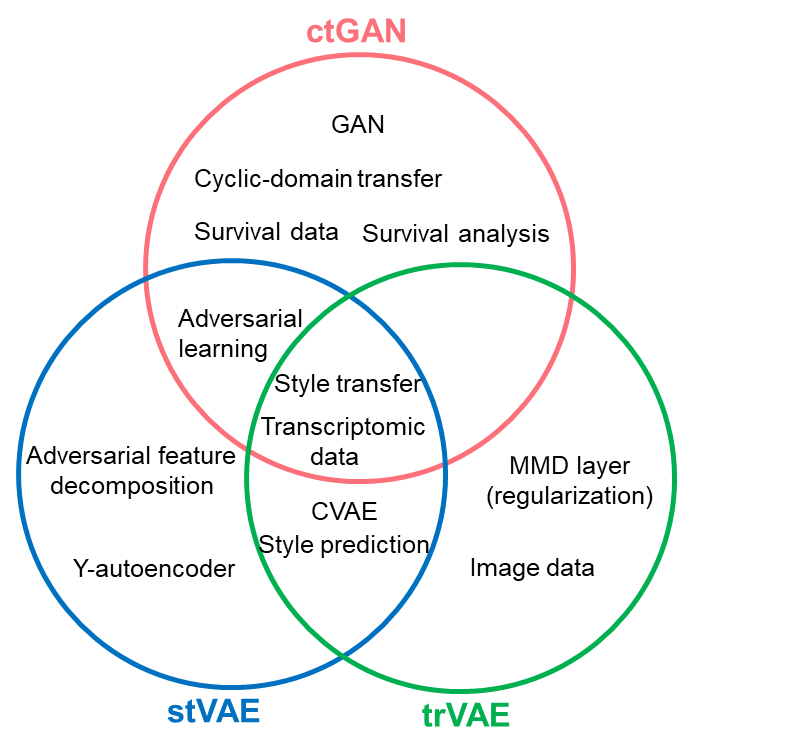
**

**Figure S6.** Venn diagram illustrating the similarities and differences between ctGAN, trVAE, and stVAE.

Figure S6 presents a Venn diagram illustrating the similarities and differences among ctGAN, trVAE, and stVAE. The common feature of these three models is their ability to generate transcriptomic data through style transformation. Both ctGAN and stVAE use adversarial learning methods, whereas both stVAE and trVAE utilize CVAE for style prediction. The distinct features of each model are as follows: ctGAN is based on a cyclic-consistent GAN model and generates both transcriptomic and survival data, enhancing survival analysis. stVAE uses an adversarial feature decomposition approach with a Y-autoencoder structure to separate style-related information from the latent vector, and trVAE incorporates an MMD layer into the decoder to match distributions across style conditions through regularization. trVAE also demonstrates performance evaluation not only on transcriptomic data but also on image data.

**Improvement in C-index using only generated data**


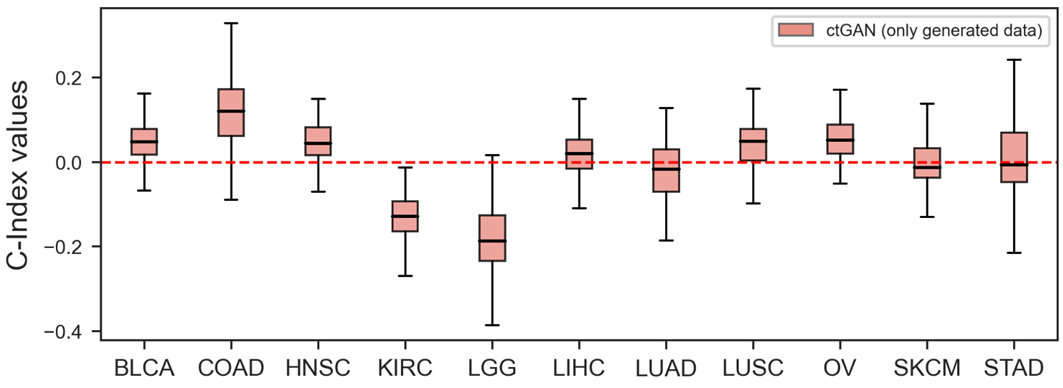


**Figure S7.** Improvement in C-index across 11 cancer types when using only generated data.

The red dashed line corresponds to the case when only real data were used. The experimental result showed an improvement in the C-index when only generated data were used. As shown in Figure S7, based on the median value obtained after 100 rounds of cross-validation, ctGAN yielded better C-index values for 6 out of the 11 cancer types considered.

The experimental results depicted in Figure 3 (main manuscript) illustrate the improvement in the C-index when both generated and real data were used together. In this case, ctGAN exhibited improved C-index values for 9 out of the 11 cancer types (excluding KIRC and LUAD). The performance significantly improved compared to using only generated data, with particularly notable enhancements observed for KIRC, LGG, LIHC, LUSC, SKCM, and STAD.
